# Ursodeoxycholic Acid Attenuates Endoplasmic Reticulum Stress-Related Retinal Pericyte Loss in Streptozotocin-Induced Diabetic Mice

**DOI:** 10.1155/2017/1763292

**Published:** 2017-01-03

**Authors:** Yoo-Ri Chung, Jeong A. Choi, Jae-Young Koh, Young Hee Yoon

**Affiliations:** ^1^Department of Ophthalmology, Asan Medical Center, University of Ulsan College of Medicine, Seoul, Republic of Korea; ^2^Neural Injury Research Center, Asan Institute for Life Sciences, Asan Medical Center, University of Ulsan College of Medicine, Seoul, Republic of Korea; ^3^Department of Neurology, Asan Medical Center, University of Ulsan College of Medicine, Seoul, Republic of Korea

## Abstract

Loss of pericytes, an early hallmark of diabetic retinopathy (DR), results in breakdown of the blood-retinal barrier. Endoplasmic reticulum (ER) stress may be involved in this process. The purpose of this study was to examine the effects of ursodeoxycholic acid (UDCA), a known ameliorator of ER stress, on pericyte loss in DR of streptozotocin- (STZ-) induced diabetic mice. To assess the extent of DR, the integrity of retinal vessels and density of retinal capillaries in STZ-induced diabetic mice were evaluated. Additionally, induction of ER stress and the unfolded protein response (UPR) were assessed in diabetic mice and human retinal pericytes exposed to advanced glycation end products (AGE) or modified low-density lipoprotein (mLDL). Fluorescein dye leakage during angiography and retinal capillary density were improved in UDCA-treated diabetic mice, compared to the nontreated diabetic group. Among the UPR markers, those involved in the protein kinase-like ER kinase (PERK) pathway were increased, while UDCA attenuated UPR in STZ-induced diabetic mice as well as AGE- or mLDL-exposed retinal pericytes in culture. Consequently, vascular integrity was improved and pericyte loss reduced in the retina of STZ-induced diabetic mice. Our findings suggest that UDCA might be effective in protecting against DR.

## 1. Introduction

Diabetic retinopathy (DR), a retinal microvascular disease, is one of the leading causes of severe vision loss among the working-age population [1, 2]. Proliferative retinopathy and diabetic macular edema are major complications of DR and lead to severe visual impairment [3]. Strict control of blood glucose as well as early detection and treatment are often effective measures in preventing severe vision loss due to DR [3]. However, the currently available treatment modalities, such as laser photocoagulation, intravitreal injection of antivascular endothelial growth factor (VEGF) agents, or surgery, are mainly focused on macular edema or late-stage DR [3].

The pathogenesis of DR is extremely complex, involving multiple mechanisms. Numerous studies have revealed apoptosis of retinal ganglion cells and inner nuclear layer degeneration in diabetic retina, suggesting that neuroretinal degeneration is an important step in DR development [4–6]. Inflammation is additionally believed to be involved in DR [7]. Our group has focused on vascular alterations, as DR is generally considered a microvascular complication of diabetes. One of the early key pathological events of DR is breakdown of the blood-retinal barrier (BRB) [1, 7]. Pericytes are essential in the maintenance of vascular integrity/BRB, and their functional abnormalities and eventual loss may play a critical role in the breakdown of BRB in DR [1, 4, 8–10]. Hyperglycemia leads to pericyte loss, either directly or through nonenzymatic formation of advanced glycation end products (AGE) [11, 12]. Modified low-density lipoprotein (mLDL), associated with accelerated atherosclerosis in diabetes, may also induce injury of retinal capillary pericytes [13–16]. Loss of pericytes subsequently results in vascular abnormalities accompanied by upregulation of angiogenic factors and inflammatory cytokines, such as VEGF and monocyte chemoattractant protein 1 (MCP-1) [1, 17].

The endoplasmic reticulum (ER) is an intracellular compartment that functions in protein biosynthesis and folding [18]. When ER function is disrupted, unfolded and misfolded proteins accumulate within the organelle, a situation termed ER stress [18, 19]. ER stress induces the unfolded protein response (UPR) in cells to restore ER homeostasis. Although UPR is regarded as a cellular mechanism triggered to overcome ER stress, it may lead to apoptosis in cases of prolonged and severe ER stress [20]. UPR begins with an increase in interactions between binding protein/glucose-regulated protein 78 (Bip/GRP78), a key ER stress regulatory chaperone protein, and unfolded proteins in the ER [21, 22]. Three signaling branches of the UPR are triggered by the dissociation of Bip/GRP78 from three integral ER membrane proteins, specifically, protein kinase-like ER kinase/eukaryotic translation initiation factor-2*α* (PERK/eIF2*α*), inositol-requiring enzyme 1 (IRE1), and activating transcription factor 6 (ATF6) [18–22]. In cases where initiation of these branch pathways cannot resolve the accumulation of unfolded proteins, apoptotic pathways are triggered [20]. The most significant apoptotic pathway in the ER is PERK/eIF2*α*- or ATF6-dependent transcriptional induction of CCAAT-enhancer-binding protein homologous protein (CHOP) [20]. UPR was initially detected in neurodegenerative disorders, such as Alzheimer's disease and Parkinson's disease. Recent reports suggest that ER stress and UPR may be associated with diabetes [22, 23]. Moreover, ER stress has been shown to contribute to retinal inflammation in DR [18, 19, 22]. Fluctuations in glucose levels that often occur in diabetes can induce ER stress in retinal pericytes, a change associated with the early stage of DR [22–25].

Ursodeoxycholic acid (UDCA) and tauroursodeoxycholic acid (TUDCA), drugs for primary biliary cirrhosis and other cholestatic disorders, act as chemical chaperones that alleviate ER stress and exert protective effects on retinal cells under conditions of hyperglycemia, retinal detachment, choroidal neovascularization, and light-induced retinal damage [26, 27]. ER stress, potentially involved in the early stage of DR, may be an ideal target for early therapeutic intervention [28]. This study was performed to investigate the role of ER stress in DR and further ascertain whether UDCA, a known ER stress alleviator, exerts a therapeutic effect in streptozotocin- (STZ-) induced diabetic mice.

## 2. Materials and Methods

### 2.1. Chemicals

STZ, UDCA, fluorescein isothiocyanate-dextran, and dimethyl sulfoxide (DMSO) were purchased from Sigma (St. Louis, MO, USA). AGE was purchased from BioVision (Milpitas, CA, USA). mLDL was prepared by diluting low-density lipoprotein (Calbiochem; La Jolla, CA, USA) in phosphate buffered saline (PBS) and incubating with 10 *μ*M CuCl_2_ at 37°C for 24 h [29, 30].

### 2.2. Animals

The ARVO Statement for the Use of Animals in Ophthalmic and Vision Research was followed, and experimental protocols were approved by the Internal Review Board for Animal Experiments of the Asan Life Science Institute, University of Ulsan College of Medicine (Seoul, Korea). C57BL/6NCrSlc male mice 8 weeks of age (22–25 g) were obtained from Japan SLC, Inc. (Hamamatsu, Japan) and maintained at 24 ± 0.5°C under a 12 h light/dark cycle. Animals were injected once intraperitoneally with STZ (150 mg/kg body weight in 50 mM citrate buffer, pH 4.5) or citrate buffer alone (control) after 4 h of fasting [31, 32]. Mice with blood glucose levels higher than 300 mg/dL were considered diabetic at 1 week after STZ injection. Two weeks after injection, the experiment was initiated by injecting animals intraperitoneally with UDCA (100 mg/kg/d body weight) or 10% cyclodextrin buffer in saline daily for 6 weeks. This dose of UDCA was selected based on earlier experiments by Woo et al. [33] and our preliminary studies. UDCA (100 mg/kg/d body weight) attenuated UPR marker expression and reduced vascular leakage in fluorescein angiography. The dose used was lower than that by Woo et al. [33].

### 2.3. Fundus Photography and Fluorescein Angiography

Fluorescein angiography was performed with the Micron III retinal imaging system (Phoenix Research Laboratories, Inc., Pleasanton, CA, USA) under inhalation anesthesia with 1.5% isoflurane at 8 weeks after STZ injection. Pathological vascular changes are reported to be diverse even within the same species of chemically induced diabetic animal models [34]. Vascular leakage was reported to appear at 2 months [35], and we performed angiography at 8 weeks in our setting. Images were captured with a contact lens in Micron III after intraperitoneal injection with 0.2 ml of 2% fluorescein sodium (Alcon Laboratories, Inc., Fort Worth, TX, USA). Fluorescein angiographs of the early phase were obtained 3 min after fluorescein injection while those of the late phase were taken at 15 min. The intensity of fluorescein leakage was quantified with ImageJ software (NIH, Bethesda, MD, USA) and averaged from 10 different measurements.

### 2.4. FITC Staining of Retinal Flat-Mount

We sacrificed mice 8 weeks after STZ injection, when we evaluated as the time-point of prominent vascular change, from our previous experiment [36]. Anesthesia was performed via intramuscular injection of 0.3 ml of Zoletil (diluted 1 : 5) and cardiac perfusion with 0.5 ml of 15 mg/ml fluorescein-dextran (2,000 kDa, #FD2000S, Sigma). After 5 min, eyes were fixed in 4% paraformaldehyde for 1 h and shifted to a culture dish filled with PBS. Retinal tissues were dissected, flat-mounted on glass slides with coverslips, and examined via confocal microscopy (Carl-Zeiss, Oberkochen, Germany). Capillary density was measured as the number of capillaries counted in a 500 *μ*m straight line, and 15 measurements of capillary density were averaged for each retina [37].

### 2.5. Immunohistochemistry

Collected retinas were fixed in 4% paraformaldehyde, washed in PBS, and incubated with permeabilizing and blocking solution, specifically, PBS containing 0.2% Triton X-100 and 1% bovine serum albumin. The presence of retinal pericytes was assessed by immunostaining retinal tissues incubated with anti-PDGFR-*β* antibody (1 : 100, Epitomics, Burlingame, CA, USA) at 4°C for 7 days and subsequently with Alexa Fluor-conjugated secondary antibodies at 4°C for 1 day (1 : 500; Alexa Fluor 555-donkey anti-rabbit IgG, Invitrogen).

### 2.6. Human Retinal Pericyte Culture

The human retinal pericyte cell line was obtained from the Applied Cell Biology Research Institute (Suite B, Kirkland, WA, USA) and cultured in Dulbecco's Modified Eagle Medium with 1 g/L glucose (Invitrogen, Carlsbad, CA, USA) supplemented with 10% fetal bovine serum (Invitrogen), 1% penicillin-streptomycin (Lonza, Allendale, NJ, USA), and 2 mM glutamine (Sigma) at 37°C in a humidified 5% CO_2_ incubator. Cells used for experiments were at ~80% confluence. Cells were exposed to either 100 *μ*g/ml AGE or 500 *μ*g/ml mLDL with or without 100 *μ*M UDCA dissolved in DMSO. mLDL prepared in PBS and incubated with CuCl_2_ was used as a control. The doses of AGE or mLDL used were based on data from a previous study by our group [36], while 100 *μ*M UDCA was the lowest concentration effective in attenuating expression of UPR markers in cultured retinal pericytes.

### 2.7. Cell Death Assay

We performed propidium iodide (PI, Sigma) staining under similar conditions as above to determine the number of apoptotic cells [38, 39]. Briefly, human retinal pericytes were plated on 24-well plates and exposed to either 100 *μ*g/ml AGE for 24 h or 500 *μ*g/ml mLDL for 12 h in 500 *μ*l medium, followed by 1 *μ*l of 1 *μ*g/ml PI dissolved in distilled water for 5 min at 37°C. Dead cells were counted under a fluorescence microscope [35].

### 2.8. Western Blot Analysis

Eyeballs were enucleated for evaluation of UPR markers at 4 weeks after STZ injection, while those for evaluation of inflammatory cytokines were harvested at 8 weeks [36]. Pericytes and whole eyeball tissues were lysed in radioimmunoprecipitation assay (RIPA) buffer (20 mM Tris-Cl pH 7.4, 150 mM NaCl, 1 mM EDTA, 1 mM EGTA, 1% Triton X-100, 2.5 mM sodium pyrophosphate, 1 *μ*M Na_3_VO_4_, 1 *μ*g/ml leupeptin, and 1 mM phenylmethylsulfonyl fluoride). Lysates were centrifuged and supernatant protein concentrations determined using the Dc Protein Assay Reagent (BioRad, Hercules, CA, USA). Samples containing equal amounts of protein were separated via sodium dodecyl sulfate-polyacrylamide gel electrophoresis (SDS-PAGE) and transferred to polyvinylidene difluoride membrane (Millipore, Bedford, MA, USA). Membranes were incubated overnight at 4°C with primary antibodies, followed by horseradish peroxidase-conjugated goat anti-rabbit IgG or anti-mouse IgG, as appropriate (1 : 10,000; Pierce, Rockford, IL, USA). The primary antibodies used were anti-GRP78 (1 : 2500; Abcam, Cambridge, MA, USA), anti-ATF6 (1 : 1000, NOVUS, Littleton, CO, USA), anti-pPERK (1 : 500, Santa Cruz Technology, Santa Cruz, CA, USA), anti-peIF2*α* (1 : 1000; Cell Signaling Technology, Danvers, MA, USA), anti-CHOP (1 : 1000, Santa Cruz), anti-MCP-1 (1 : 1000; NOVUS), anti-p-TNF*α* (1 : 1000; R&D Systems, Minneapolis, MN, USA), and anti-*β*-actin (1 : 2500; Sigma). Protein band intensities were quantified by densitometry using ImageJ software.

### 2.9. Statistical Analysis

All results are presented as means ± SEM. Statistical tests were performed using SPSS version 23.0 for Windows (SPSS Inc., Chicago, IL). Student's *t*-test or Mann–Whitney *U* test was used to evaluate the significance of differences between groups, and *P* values < 0.05 were considered significant. All statistical analyses and graphical presentations were conducted using Sigma Plot version 10.0 software.

## 3. Results

### 3.1. Effect of UDCA on Vascular Integrity in STZ-Induced Diabetic Mice

**Figure 1:**
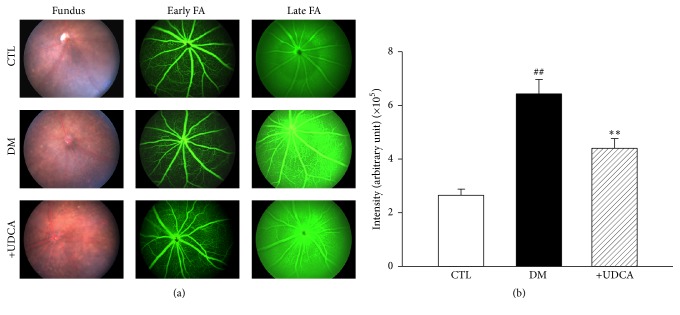
Effect of UDCA on vascular integrity in STZ-induced diabetic mice. (a) Representative fundus images and fluorescein angiography (FA) of early- and late-phase DR in control nontreated and UDCA-treated diabetic mice. (b) Fluorescein leakage was increased in diabetic mice (*n* = 8) at the late phase of fluorescein angiography, compared to control mice (*n* = 8), and decreased in diabetic mice treated with 100 mg/kg UDCA (*n* = 11). Fluorescein angiographs of the early phase were obtained 3 min after fluorescein injection (early FA), while those of the late phase (late FA) were taken at 15 min. ^##^*P* < 0.001 versus control mice, ^*∗∗*^*P* < 0.01 versus nontreated diabetic mice.

**Figure 2:**
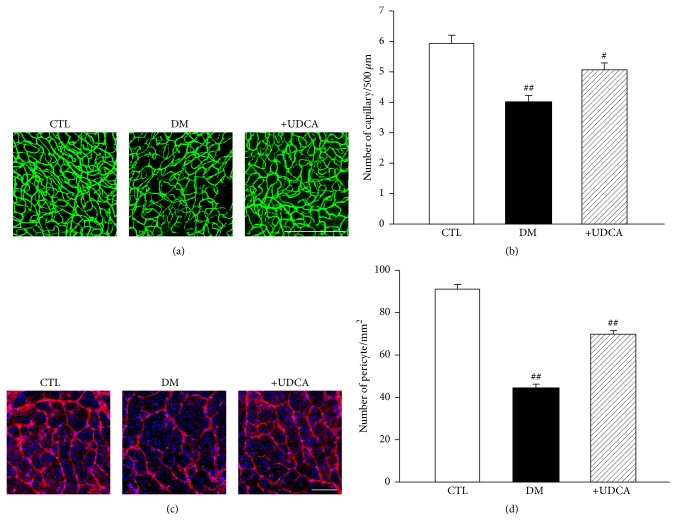
Effect of UDCA on pericyte loss in STZ-induced diabetic mice. (a) Representative images of FITC staining in control, nontreated diabetic, and UDCA-treated diabetic mice. Original magnification: ×200; scale bar: 500 *μ*m. (b) Capillary density was lower in nontreated diabetic mice, while no differences were observed between UDCA-treated diabetic and control mice. Fifteen measures of capillary density were averaged for each retina from control (*n* = 5), nontreated diabetic mice (*n* = 5), and UDCA-treated diabetic mice (*n* = 5). ^##^*P* < 0.001 versus control mice, ^#^*P* < 0.005 versus nontreated diabetic mice. (c) Representative images of PDGFR-*β* staining for retinal pericytes in control, nontreated diabetic and UDCA-treated diabetic mice. Original magnification: ×200; scale bar: 200 *μ*m. (d) The number of pericytes was lower in nontreated diabetic mice while no differences between UDCA-treated diabetic and control mice were evident. Measurements of pericyte number were averaged for each retina from control (*n* = 5), nontreated diabetic mice (*n* = 5), and UDCA-treated diabetic mice (*n* = 6). CTL: control mice, DM: nontreated diabetic mice, and +UDCA: diabetic mice treated with UDCA, ^##^*P* < 0.001 versus control mice.

Leakage of fluorescein dye occurs in cases of increased capillary permeability and is considered a marker for BRB breakdown in DR [12]. Using fluorescein angiography obtained with a retinal imaging system for living mice (Micron III), we examined the effect of UDCA on vascular integrity in diabetic mice. UDCA treatment was initiated 2 weeks after STZ injection and then continued for 6 weeks. Increased fluorescein leakage was observed at a late phase in retinas of STZ diabetic mice, compared with controls, which was significantly attenuated upon UDCA treatment (Figures 1(a) and 1(b)). To evaluate the effect of UDCA on retinal microvasculature in diabetic mice, we measured the linear capillary density by counting the number of capillaries intersected by a 500 *μ*m line on fluorescein isothiocyanate (FITC) images at 8 weeks after STZ injection [36, 37]. Linear capillary density was decreased in nontreated diabetic mice, compared with controls, but not in UDCA-treated mice (Figures 2(a) and 2(b)). Immunostaining with anti-platelet-derived growth factor receptor-*β* (PDGFR-*β*) antibody specific to pericytes showed loss of retinal pericytes in retinal whole flat mounts of nontreated diabetic mice, which was protected in UDCA-treated mice (Figures 2(c) and 2(d)).

### 3.2. Attenuation of UPR and Inflammatory Cytokine Induction in Diabetic Mice by UDCA

**Figure 3:**
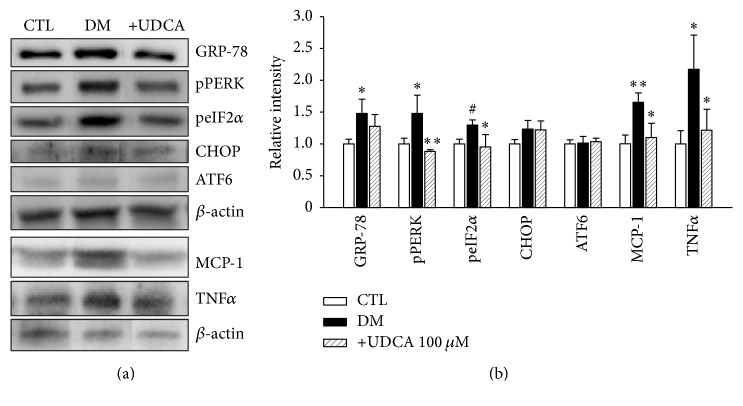
Induction of UPR and inflammatory cytokines and UDCA-mediated attenuation in diabetic mice. Levels of UPR markers and inflammatory cytokines were assessed via western blot analysis of eye tissue from control (*n* = 5), nontreated DM (*n* = 5), and UDCA-treated DM (*n* = 5) groups. (a) Representative results of western blot analyses are shown. (b) Increased levels of GRP78, pPERK, peIF2*α*, MCP-1, and TNF *α* in diabetic mice, compared to the control group, were attenuated by UDCA. ATF6 and CHOP expression was not significantly different among groups. CTL: control mice, DM: nontreated diabetic mice, and +UDCA: diabetic mice treated with UDCA, ^*∗*^*P* < 0.05, ^*∗∗*^*P* < 0.01, ^#^*P* < 0.005.

**Figure 4:**
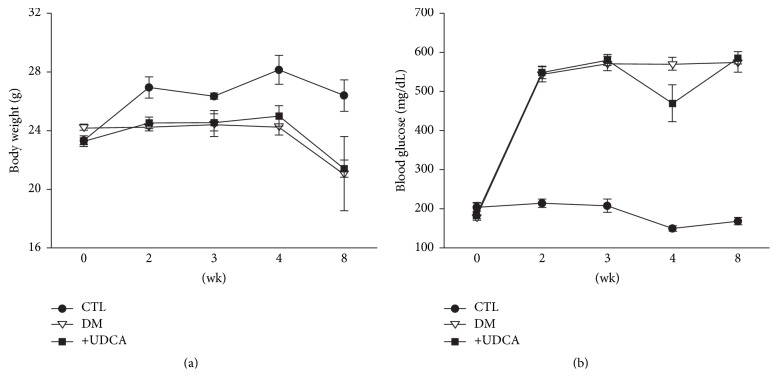
Body weights and blood glucose levels throughout the experimental period. No significant differences were evident in body weight (a) or blood glucose levels (b) between control and diabetic mouse groups with or without UDCA treatment. CTL: control mice, DM: nontreated diabetic mice, and +UDCA: diabetic mice treated with UDCA.

Based on the recent finding that ER stress is involved in early-stage DR, we performed western blot of eye tissue harvested 2 to 6 weeks after STZ injection. Expression of UPR markers began to increase at 3 weeks, peaked at 4 weeks, and decreased at 6 weeks after STZ injection (data not shown). Based on these data, we selected 4 weeks showing prominent expression of UPR as the time-point to examine the effects of UDCA on UPR markers. As shown in Figure 3, expression levels of GRP78, pPERK, and peIF2*α* were elevated in retinal tissues of diabetic mice, which were attenuated following UDCA treatment. However, ATF6 and CHOP displayed no significant alterations in expression patterns. Concurrent with the increased expression of specific UPR markers, levels of MCP-1, and tumor necrosis factor *α* (TNF-*α*), cytokines associated with diabetic retinopathy were elevated. Notably, these increases were attenuated upon UDCA treatment. To eliminate the possibility that UDCA induces better control of blood glucose levels to exert a protective effect against DR, body weight and blood glucose levels in all three groups of mice were measured throughout the experimental period. These metabolic parameters were not markedly different among the control, diabetic, and UDCA-treated diabetic groups (Figure 4).

### 3.3. UDCA Attenuates Induction of UPR and Inflammatory Cytokines in Retinal Pericytes

**Figure 5:**
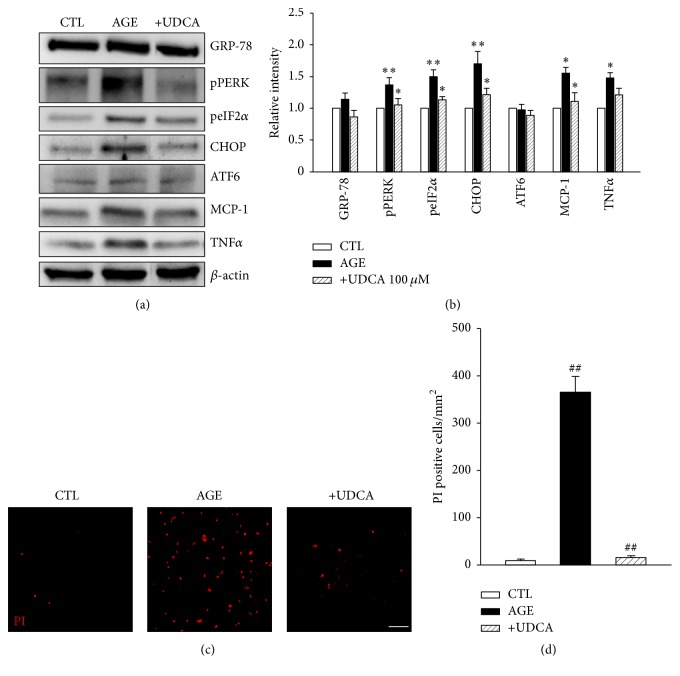
AGE-induced UPR, inflammatory cytokine expression, and cell death are attenuated by UDCA in retinal pericytes. (a) Typical results of five independent western blots are shown. (b) Increase in pPERK, peIF2*α*, CHOP, and MCP-1 was attenuated upon cotreatment with UDCA. GRP78, ATF6, and TNF-*α* did not display significant changes in expression among groups. (c, d) PI staining demonstrating a protective effect of UDCA against cell death in AGE-exposed retinal pericytes. Original magnification: ×100; scale bar: 100 *μ*m. CTL: control pericytes, AGE: AGE-exposed pericytes, and +UDCA: AGE-exposed pericytes cotreated with UDCA, ^*∗*^*P* < 0.05, ^*∗∗*^*P* < 0.01, ^##^*P* < 0.001.

**Figure 6:**
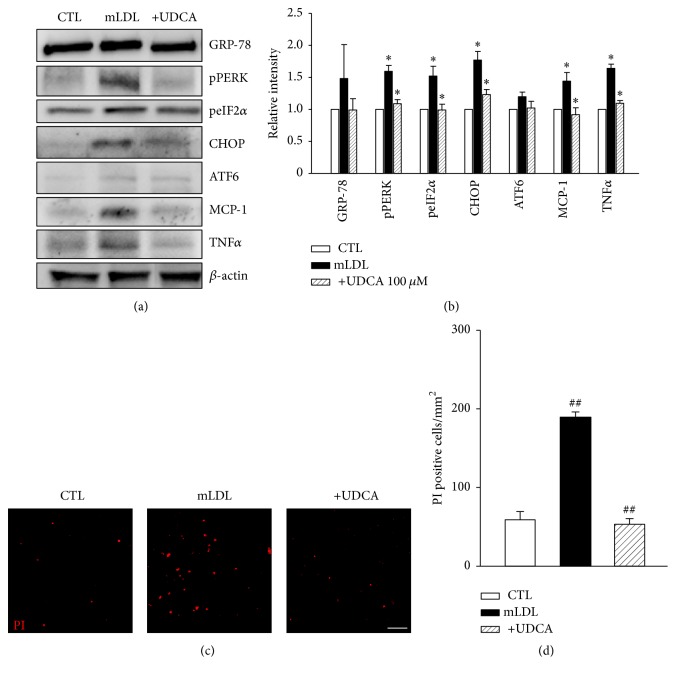
mLDL-induced UPR, inflammatory cytokine expression, and cell death are attenuated by UDCA in retinal pericytes. (a) Typical results of four independent western analyses are shown. (b) Levels of pPERK, peIF2*α*, CHOP, and MCP-1 were increased and attenuated upon cotreatment with UDCA. GRP78, ATF6, and TNF-*α* did not display significant changes in expression. (c, d) PI staining demonstrating the protective effect of UDCA against cell death in mLDL-exposed retinal pericytes. Original magnification: ×100; scale bar: 100 *μ*m. CTL: control pericytes, mLDL: mLDL-exposed pericytes, and +UDCA: mLDL-exposed pericytes cotreated with UDCA, ^*∗*^*P* < 0.05, ^##^*P* < 0.001.

Hyperglycemia and dyslipidemia, considered systemic risk factors in DR [12], may act through multiple pathways. One hyperglycemia-induced factor is AGE, which exerts toxic effects in cells. For lipid metabolites, modified LDL may be a significant toxic factor. Accordingly, we examined the effects of AGE and mLDL on cultured retinal pericytes [8, 15, 40, 41]. First, to determine the effects of UDCA in vitro, western blot analysis for UPR markers was performed in AGE- or mLDL-exposed human retinal pericytes. Cells were treated with either 500 *μ*g/ml AGE or 100 *μ*g/ml mLDL for 24 or 12 h, respectively, with or without cotreatment with 100 *μ*M UDCA. Notably, expression levels of pPERK, peIF2*α*, and CHOP were increased in both AGE (Figures 5(a) and 5(b)) and mLDL-treated groups (Figures 6(a) and 6(b)). These increases in expression were suppressed upon cotreatment with UDCA. In the STZ model of diabetes in mice, neither AGE nor mLDL affected the levels of GRP78 or ATF6. In addition, consistent with in vivo findings, MCP-1 and TNF-*α* levels in pericyte cultures were increased by AGE or mLDL and suppressed upon UDCA cotreatment (Figures 5 and 6). Finally, we examined the effects of UDCA on cell death induced by AGE or mLDL. The number of PI-positive dead cells was increased following exposure to AGE (Figures 5(c) and 5(d)) or mLDL (Figures 6(c) and 6(d)). Cotreatment with UDCA attenuated AGE- and mLDL-induced pericyte cell death.

## 4. Discussion

The key findings of the present study are that ER stress and inflammation occur early in the course of DR in STZ-treated diabetic mice, which are effectively suppressed by UDCA. UDCA is already in clinical use for biliary disease and may therefore be relatively quickly introduced as a new drug for DR once its efficacy is established.

DR, the most common microvascular complication of diabetes, is one of the leading causes of blindness [42, 43]. The global prevalence of diabetes is increasing, with numbers expected to reach 300 million by 2025 [44]. Recently, the global prevalence of DR was reported as ~35%. The World Health Organization has estimated that DR is responsible for 15–17% total blindness in the United States and Europe [2, 42, 43, 45]. Early clinical manifestations of DR include loss of retinal pericytes and a disrupted BRB, resulting in microaneurysms, hard exudates, and retinal hemorrhage [44, 45]. Microvascular dysfunction in DR triggers capillary hyperpermeability and occlusion, leading to macular edema and retinal neovascularization [3, 45]. Multiple randomized controlled trials have reported that intensive control of hyperglycemia and hypertension can inhibit the progression of DR, but the sight-threatening complications at the late stages remain responsible for the significant socioeconomic burden and personal costs [2, 46, 47]. The mainstay of current DR therapy is focused on vascular complications, such as diabetic macular edema or retinal neovascularization at the late stage, and is not effective in restoring previously impaired vision [48].

The ER is an intracellular organelle responsible for protein biosynthesis, folding, and maturation [18, 20]. Since the ER also acts as a sensor of environmental changes and cellular stress, various pathological conditions can compromise its protein folding capacity, resulting in accumulation of unfolded or misfolded proteins (a condition known as ER stress) [20]. The UPR is consequently activated to eliminate unfolded or misfolded proteins and restore cellular homeostasis. However, prolongation of the UPR has been shown to induce apoptosis [20, 23]. Recent studies on the molecular mechanisms of DR suggest that ER stress plays important roles in its pathogenesis [49]. Furthermore, ER stress is reported to be associated with diabetes-induced inflammation [18, 22, 25]. Multiple ER stress markers, including GRP78, p-IRE1*α*, and peIF2*α*, are upregulated in the retina of Akita mice, a genetic model of type 1 diabetes [18]. Zhong et al. [25] demonstrated increased secretion of MCP-1 and expression of ATF4 and CHOP in human retinal pericytes during intermittent hyperglycemia, a similar condition of glucose fluctuation in diabetic patients. Data from the present study showed that expression levels of UPR markers, in particular pPERK and peIF2*α* (signals involved in the PERK/eIF2*α* branch pathway), chemokines, and cytokines, such as MCP-1 and TNF*α*, are significantly increased in diabetic mice at time-points before the prominent manifestation of retinopathy.

Pericytes are essential for the integrity of retinal capillaries and regulation of capillary diameter and blood flow, and their loss is a characteristic feature in the early events of DR [1, 9, 17]. Clinically, diffuse leakage of fluorescein from hyperpermeable nascent vessels in fluorescein angiography typical of late-stage DR was consistently demonstrated in our experiments with STZ-induced diabetic mice using a mouse-specific FA imaging system. STZ-induced diabetic mice models seem to present pathological characteristics of various ranges [34, 35]. As vascular changes began to appear since 6 weeks after STZ injection [37], we observed retinal vascular changes at 8 weeks. AGE and mLDL were used, which specifically induced MCP-1 in human retinal pericytes among the several diabetes-involved cytokines examined previously [36]. While several studies reported an induction of UPR in cultured retinal pericytes under either hyperglycemic or hypoglycemic condition, [23, 50, 51], our preliminary experiments using various concentrations of glucose could not induce cytotoxic changes in cultured retinal pericytes. Instead, we were able to reproduce UPR induction with using AGE and mLDL, which are end products formed from hyperglycemia, as reported in previous studies [13, 15, 52]. Under these conditions, peIF2*α* and CHOP levels were specifically increased, supporting the significance of the PERK/eIF2*α* pathway in DR.

UDCA, an FDA-approved drug for primary biliary cirrhosis, is a chemical chaperone alleviating ER stress [53]. Protective effects of UDCA have been reported in multiple retinal diseases, such as retinal detachment, choroidal neovascularization, and light-induced retinal damage [26, 33]. Accordingly, we hypothesized that UDCA may exert protective effects via modulating ER stress in DR [28]. The mouse-specific imaging system (Micron III) was used to compare severity of retinopathy between experimental groups, as fluorescein dye leakage is an indicator of BRB loss [54]. Our experiments disclosed decreased leakage in the UDCA-treated group, compared to extensive leakage in nontreated diabetic mice, especially at the late stage of fluorescein angiography. This finding was consistent with the preserved retinal capillary density and number of retinal pericytes observed following UDCA treatment, suggesting a protective role of UDCA in vascular integrity. While some studies using* db/db* or* ob/ob* type 2 diabetic mice reported that UDCA treatment induced normalization of hyperglycemia, resulting in the protective effect on vascular change [53, 55], UDCA treatment did not normalize hyperglycemia in our STZ-induced diabetic mice. We speculate that the different method of inducing diabetes in animal models could result in this discrepancy. Attenuated cell death with UDCA cotreatment in AGE- and mLDL-exposed retinal pericytes further supports the protective role of UDCA. Moreover, this protective effect may be associated with attenuation of ER stress and diabetes-related cytokines, both in vivo and in vitro.

## 5. Conclusions

Here, we demonstrated that ER stress and UPR markers are increased at early stages in STZ-induced diabetes in mice and AGE or mLDL-exposed retinal pericytes. UDCA clearly attenuates the increase in ER stress and prevents loss of pericytes and vascular integrity in DR. Our results suggest that induction of ER stress is involved in early DR. Further research is warranted to evaluate the utility of UDCA as a therapeutic agent for DR.

## References

[1] Praidou A., Androudi S., Brazitikos P., Karakiulakis G., Papakonstantinou E., Dimitrakos S. (2010). Angiogenic growth factors and their inhibitors in diabetic retinopathy. *Current Diabetes Reviews*.

[2] Ruta L. M., Magliano D. J., Lemesurier R., Taylor H. R., Zimmet P. Z., Shaw J. E. (2013). Prevalence of diabetic retinopathy in Type 2 diabetes in developing and developed countries. *Diabetic medicine*.

[3] Bandello F., Lattanzio R., Zucchiatti I., Del Turco C. (2013). Pathophysiology and treatment of diabetic retinopathy. *Acta Diabetologica*.

[4] Eisma J. H., Dulle J. E., Fort P. E. (2015). Current knowledge on diabetic retinopathy from human donor tissues. *World Journal of Diabetes*.

[5] Chhablani J., Sharma A., Goud A. (2015). Neurodegeneration in type 2 diabetes: evidence from spectral-domain optical coherence tomography. *Investigative Ophthalmology & Visual Science*.

[6] Yang Q., Xu Y., Xie P. (2015). Retinal neurodegeneration in db/db mice at the early period of diabetes. *Journal of Ophthalmology*.

[7] Tang J., Kern T. S. (2011). Inflammation in diabetic retinopathy. *Progress in Retinal and Eye Research*.

[8] Fu D., Wu M., Zhang J. (2012). Mechanisms of modified LDL-Induced pericyte loss and retinal injury in diabetic retinopathy. *Diabetologia*.

[9] Hammes H.-P. (2005). Pericytes and the pathogenesis of diabetic retinopathy. *Hormone and Metabolic Research*.

[10] Ejaz S. (2008). Importance of pericytes and mechanisms of pericyte loss during diabetes retinopathy. *Diabetes, Obesity & Metabolism*.

[11] Pfister F., Feng Y., Hagen F. V. (2008). Pericyte migration: a novel mechanism of pericyte loss in experimental diabetic retinopathy. *Diabetes*.

[12] Klaassen I., Van Noorden C. J. F., Schlingemann R. O. (2013). Molecular basis of the inner blood-retinal barrier and its breakdown in diabetic macular edema and other pathological conditions. *Progress in Retinal and Eye Research*.

[13] Zhang S. X., Wang J. J., Dashti A. (2008). Pigment epithelium-derived factor mitigates inflammation and oxidative stress in retinal pericytes exposed to oxidized low-density lipoprotein. *Journal of Molecular Endocrinology*.

[14] Renier G., Mamputu J.-C., Desfaits A.-C., Serri O. (2003). Monocyte adhesion in diabetic angiopathy: effects of free-radical scavenging. *Journal of Diabetes and its Complications*.

[15] Sonoki K., Yoshinari M., Iwase M. (2002). Glycoxidized low-density lipoprotein enhances monocyte chemoattractant protein-1 mRNA expression in human umbilical vein endothelial cells: relation to lysophosphatidylcholine contents and inhibition by nitric oxide donor. *Metabolism: Clinical and Experimental*.

[16] Fu D., Yu J. Y., Wu M. (2014). Immune complex formation in human diabetic retina enhances toxicity of oxidized LDL towards retinal capillary pericytes. *Journal of Lipid Research*.

[17] Motiejunaite R., Kazlauskas A. (2008). Pericytes and ocular diseases. *Experimental Eye Research*.

[18] Li J., Wang J. J., Yu Q., Wang M., Zhang S. X. (2009). Endoplasmic reticulum stress is implicated in retinal inflammation and diabetic retinopathy. *FEBS Letters*.

[19] Oshitari T., Hata N., Yamamoto S. (2008). Endoplasmic reticulum stress and diabetic retinopathy. *Vascular Health and Risk Management*.

[20] Jing G., Wang J. J., Zhang S. X. (2012). ER stress and apoptosis: a new mechanism for retinal cell death. *Experimental Diabetes Research*.

[21] Smith S. S., Ryan S. J., Schachat A. P., Wilkinson C. P., Hinton D. R., Sadda S. R., Wiedemann P. (2013). Mechanisms of ER stress in retinal diseases. *Retina*.

[22] Zhang S. X., Sanders E., Wang J. J. (2011). Endoplasmic reticulum stress and inflammation: mechanisms and implications in diabetic retinopathy. *Journal of Ocular Biology, Diseases, and Informatics*.

[23] Ikesugi K., Mulhern M. L., Madson C. J. (2006). Induction of endoplasmic reticulum stress in retinal pericytes by glucose deprivation. *Current Eye Research*.

[24] Schröder M., Kaufman R. J. (2005). The mammalian unfolded protein response. *Annual Review of Biochemistry*.

[25] Zhong Y., Wang J. J., Zhang S. X. (2012). Intermittent but not constant high glucose induces ER stress and inflammation in human retinal pericytes. *Advances in Experimental Medicine and Biology*.

[26] Mantopoulos D., Murakami Y., Comander J. (2011). Tauroursodeoxycholic acid (TUDCA) protects photoreceptors from cell death after experimental retinal detachment. *PLOS ONE*.

[27] Gaspar J. M., Martins A., Cruz R., Rodrigues C. M. P., Ambrósio A. F., Santiago A. R. (2013). Tauroursodeoxycholic acid protects retinal neural cells from cell death induced by prolonged exposure to elevated glucose. *Neuroscience*.

[28] Li B., Wang H. S., Li G. G., Zhao M. J., Zhao M. H. (2011). The role of endoplasmic reticulum stress in the early stage of diabetic retinopathy. *Acta Diabetologica*.

[29] Jenkins A. J., Velarde V., Klein R. L. (2000). Native and modified LDL activate extracellular signal-regulated kinases in mesangial cells. *Diabetes*.

[30] Lyons T. J., Li W., Wojciechowski B., Wells-Knecht M. C., Wells-Knecht K. J., Jenkins A. J. (2000). Aminoguanidine and the effects of modified LDL on cultured retinal capillary cells. *Investigative Ophthalmology and Visual Science*.

[31] Tesch G. H., Allen T. J. (2007). Rodent models of streptozotocin-induced diabetic nephropathy (methods in renal research). *Nephrology*.

[32] Furman B. L. (2015). Streptozotocin-induced diabetic models in mice and rats. *Current Protocols in Pharmacology*.

[33] Woo S. J., Kim J. H., Yu H. G. (2010). Ursodeoxycholic acid and tauroursodeoxycholic acid suppress choroidal neovascularization in a laser-treated rat model. *Journal of Ocular Pharmacology and Therapeutics*.

[34] Cai X., McGinnis J. F. (2016). Diabetic retinopathy: animal models, therapies, and perspectives. *Journal of Diabetes Research*.

[35] Lai A. K. W., Lo A. C. Y. (2013). Animal models of diabetic retinopathy: summary and comparison. *Journal of Diabetes Research*.

[36] Choi J. A. (2013). *Anti-ALS drus riluzole attenuates pericyte loss in diabetic retinopathy of streptozotocin-treated mouse [M.S. thesis]*.

[37] Leskova W., Watts M. N., Carter P. R., Eshaq R. S., Harris N. R. (2013). Measurement of retinal blood flow rate in diabetic rats: disparity between techniques due to redistribution of flow. *Investigative Ophthalmology & Visual Science*.

[38] Newbold A., Martin B. P., Cullinane C., Bots M. (2014). Detection of apoptotic cells using propidium iodide staining. *Cold Spring Harbor Protocols*.

[39] Riccardi C., Nicoletti I. (2006). Analysis of apoptosis by propidium iodide staining and flow cytometry. *Nature Protocols*.

[40] Chew E. Y., Davis M. D., Danis R. P. (2014). The effects of medical management on the progression of diabetic retinopathy in persons with type 2 diabetes: the Action to Control Cardiovascular Risk in Diabetes (ACCORD) Eye Study. *Ophthalmology*.

[41] Du M., Wu M., Fu D. (2013). Effects of modified LDL and HDL on retinal pigment epithelial cells: a role in diabetic retinopathy?. *Diabetologia*.

[42] Yau J. W. Y., Rogers S. L., Kawasaki R. (2012). Global prevalence and major risk factors of diabetic retinopathy. *Diabetes Care*.

[43] Stitt A. W., Lois N., Medina R. J., Adamson P., Curtis T. M. (2013). Advances in our understanding of diabetic retinopathy. *Clinical Science*.

[44] Williams R., Airey M., Baxter H., Forrester J., Kennedy-Martin T., Girach A. (2004). Epidemiology of diabetic retinopathy and macular oedema: a systematic review. *Eye*.

[45] Resnikoff S., Pascolini D., Etya'ale D. (2004). Global data on visual impairment in the year 2002. *Bulletin of the World Health Organization*.

[46] Chew E. Y., Ambrosius W. T., Davis M. D. (2010). Effects of medical therapies on retinopathy progression in type 2 diabetes. *The New England Journal of Medicine*.

[47] The Diabetes Control and Complications Trial (1995). The effect of intensive diabetes treatment on the progression of diabetic retinopathy in insulin-dependent diabetes mellitus. *Archives of Ophthalmology*.

[48] Aiello L. P. (2002). The potential role of PKC *β* in diabetic retinopathy and macular edema. *Survey of Ophthalmology*.

[49] Hu W.-K., Liu R., Pei H., Li B. (2012). Endoplasmic reticulum stress-related factors protect against diabetic retinopathy. *Experimental Diabetes Research*.

[50] Shin E. S., Huang Q., Gurel Z. (2014). STAT1-mediated Bim expression promotes the apoptosis of retinal pericytes under high glucose conditions. *Cell Death & Disease*.

[51] Devi T. S., Hosoya K.-I., Terasaki T., Singh L. P. (2013). Critical role of TXNIP in oxidative stress, DNA damage and retinal pericyte apoptosis under high glucose: implications for diabetic retinopathy. *Experimental Cell Research*.

[52] Chen B.-H., Jiang D.-Y., Tang L.-S. (2006). Advanced glycation end-products induce apoptosis involving the signaling pathways of oxidative stress in bovine retinal pericytes. *Life Sciences*.

[53] Özcan U., Yilmaz E., Özcan L. (2006). Chemical chaperones reduce ER stress and restore glucose homeostasis in a mouse model of type 2 diabetes. *Science*.

[54] Wisniewska-Kruk J., Klaassen I., Vogels I. M. C. (2014). Molecular analysis of blood-retinal barrier loss in the Akimba mouse, a model of advanced diabetic retinopathy. *Experimental Eye Research*.

[55] Cao A.-L., Wang L., Chen X. (2016). Ursodeoxycholic acid and 4-phenylbutyrate prevent endoplasmic reticulum stress-induced podocyte apoptosis in diabetic nephropathy. *Laboratory Investigation*.

