# Corrigendum to “Ursodeoxycholic Acid Attenuates Endoplasmic Reticulum Stress-Related Retinal Pericyte Loss in Streptozotocin-Induced Diabetic Mice”

**DOI:** 10.1155/2024/9809651

**Published:** 2024-07-17

**Authors:** Yoo-Ri Chung, Jeong A. Choi, Jae-Young Koh, Young Hee Yoon

**Affiliations:** ^1^ Department of Ophthalmology Asan Medical Center University of Ulsan College of Medicine, Seoul, Republic of Korea; ^2^ Neural Injury Research Center Asan Institute for Life Sciences Asan Medical Center University of Ulsan College of Medicine, Seoul, Republic of Korea; ^3^ Department of Neurology Asan Medical Center University of Ulsan College of Medicine, Seoul, Republic of Korea

In the article titled “Ursodeoxycholic Acid Attenuates Endoplasmic Reticulum Stress-Related Retinal Pericyte Loss in Streptozotocin-Induced Diabetic Mice” [1], discontinuities in Figures 3(a), 5(a), and 6(a) were identified, as raised on PubPeer [2]. The authors have explained that the discontinuities are a result of their image composition process. They apologise for the confusion caused and provide an explanation of their process, as seen below. The authors would also like to update Section 2.8 of the article to include a more complete description of the image processing method used, shown below, and have provided their raw data as supporting information.

Explanation of image processing:

In our Western blot analysis shown in Figure 3(a), we ran a total of 12 lanes of proteins from the three experimental groups (CTL vs. DM vs. UDCA). Then, in order to improve the readability and presentation of the figures, we chose one representative blot from each group whose intensity was the closest to the mean value of each group and spliced them as shown in Figure 3(a). The densitometry data shown in Figure 3(b) were calculated as the mean of the intensities of all blots from each group. Similarly, the blots in Figures 5(a) and 6(a) were cropped from whole blots (provided in the Supporting Information (available here)) in which samples with different drug concentrations were run simultaneously. Of those, we chose to present the 100 *μ*M UDCA blots because they showed the most significant change from the AGE group. The densitometry data of the blots are shown in Figures 5(b) and 6(b), respectively.

The Western blot images used in this study were uniformly acquired and composed according to the following process. Enhanced chemiluminescence (ECL) was performed using the Autochemi system (UPS), and chemiluminescent signals were captured using LABWORK software and a camera. The blots were detected using Immobilon® Western HRP substrate (Millipore, USA). The blot images were acquired at 60 cuts per minute, and each blot image was exported to Adobe Photoshop. The brightness and contrast of the whole blot were adjusted to enhance readability. The brightness/contrast was adjusted as a whole in each blot, and those of individual lanes were not separately adjusted. Then, the protein bands whose intensities were the closest to the mean value of each group (quantified using the ImageJ software) were selected and composed into final figures by cut-and-paste using Adobe Photoshop.

The corrected Section 2.8 should read:

## 2.8. Western Blot Analysis Section

Eyeballs were enucleated for evaluation of UPR markers at 4 weeks after STZ injection, while those for evaluation of inflammatory cytokines were harvested at 8 weeks [36]. Pericytes and whole eyeball tissues were lysed in radioimmunoprecipitation assay (RIPA) buffer (20 mM Tris-Cl pH 7.4, 150 mM NaCl, 1 mM EDTA, 1 mM EGTA, 1% Triton X-100, 2.5 mM sodium pyrophosphate, 1 *μ*M Na3VO4, 1 *μ*g/mL leupeptin, and 1 mM phenylmethylsulfonyl fluoride). Lysates were centrifuged, and supernatant protein concentrations were determined using the DC Protein Assay Reagent (BioRad, Hercules, CA, USA). Samples containing equal amounts of protein were separated via sodium dodecyl sulfate-polyacrylamide gel electrophoresis (SDS-PAGE) and transferred to a polyvinylidene difluoride membrane (Millipore, Bedford, MA, USA). Membranes were incubated overnight at 4°C with primary antibodies, followed by horseradish peroxidase-conjugated goat anti-rabbit IgG or anti-mouse IgG, as appropriate (1:10,000; Pierce, Rockford, IL, USA). The primary antibodies used were anti-GRP78 (1:2500; Abcam, Cambridge, MA, USA), anti-ATF6 (1:1000; NOVUS, Littleton, CO, USA), anti-pPERK (1:500; Santa Cruz Technology, Santa Cruz, CA, USA), anti-peIF2*α* (1:1000; Cell Signaling Technology, Danvers, MA, USA), anti-CHOP (1:1000; Santa Cruz), anti-MCP-1 (1:1000; NOVUS), anti-p-TNF*α* (1:1000; R&D Systems, Minneapolis, MN, USA), and anti-*β*-actin (1:2500; Sigma). Protein band intensities were quantified by densitometry using ImageJ software.

ECL was performed using the UPS, and chemiluminescent signals were captured using LABWORK software and a camera. The blots were detected using Immobilon® Western HRP substrate (Millipore, USA). The blot images were acquired by 60 cuts per minute, and each blot image was exported to Adobe Photoshop, and the brightness and contrast of the whole blot were adjusted to enhance the readability. The brightness/contrast was adjusted as a whole in each blot, and those of individual lanes were not separately adjusted. Then, the protein bands whose intensities were the closest to the mean value of each group (quantified using the ImageJ software) were selected and composed into final figures by cut-and-paste using Adobe Photoshop.

## Supporting Information

Supporting Information Additional supporting information can be found online in the Supporting Information section. The authors provided the original figures and data in a high-resolution format, which are included as supporting information.
