# Supplementary figures and images for: Corrigendum to “Ursodeoxycholic Acid Attenuates Endoplasmic Reticulum Stress-Related Retinal Pericyte Loss in Streptozotocin-Induced Diabetic Mice”
Source: J Diabetes Res. 2024 Jul 17;2024:9809651. doi: 10.1155/2024/9809651 (PMC11268973; doi:10.1155/2024/9809651)

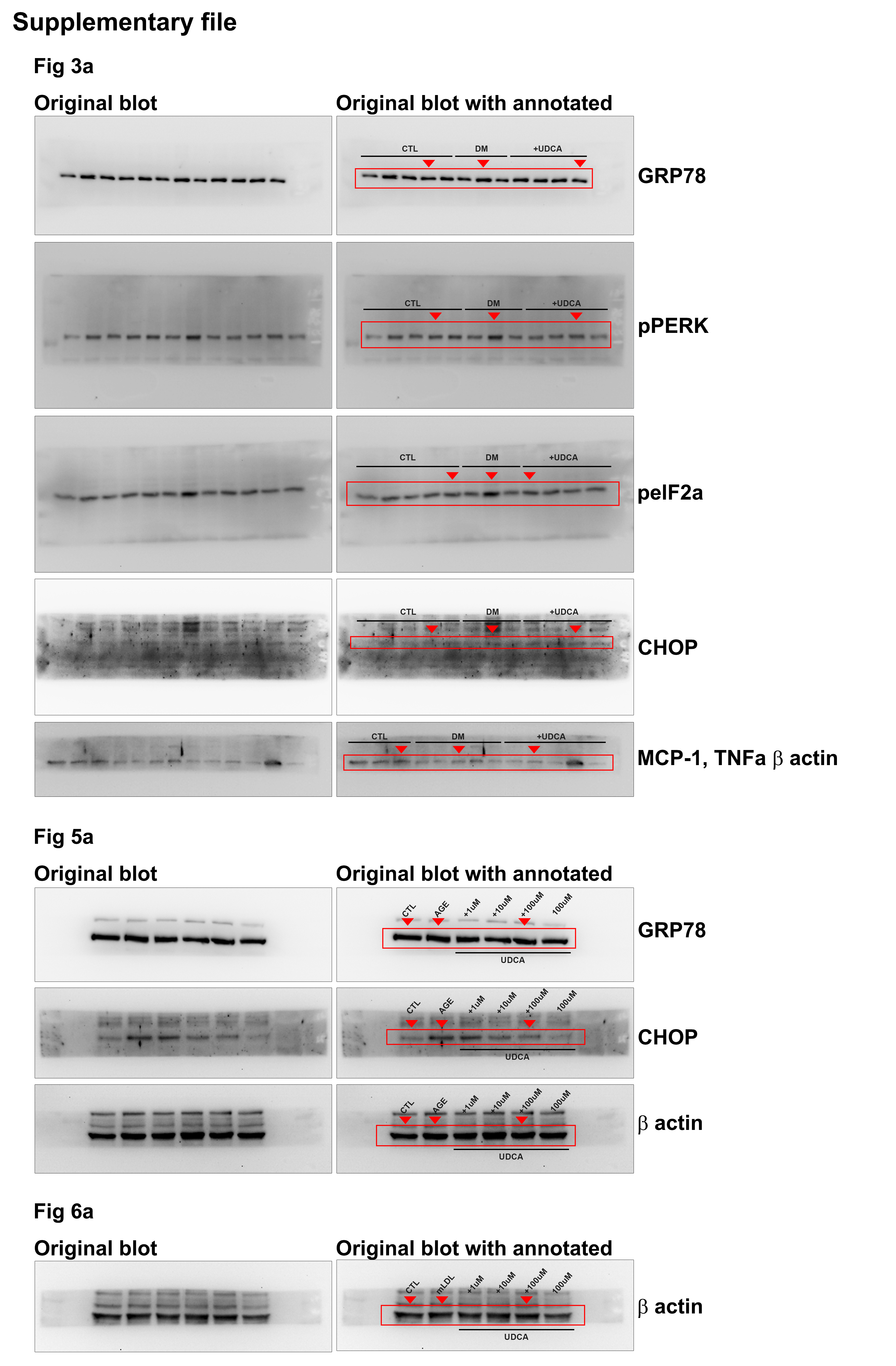

Supplement: Supporting Information — Additional supporting information can be found online in the Supporting Information section. The authors provided the original figures and data in a high-resolution format, which are included as supporting information. [file 9809651.f2.tif]
